# Time-resolved HAXPES using a microfocused XFEL beam: From vacuum space-charge effects to intrinsic charge-carrier recombination dynamics

**DOI:** 10.1038/srep35087

**Published:** 2016-10-12

**Authors:** Lars-Philip Oloff, Ashish Chainani, Masaharu Matsunami, Kazutoshi Takahashi, Tadashi Togashi, Hitoshi Osawa, Kerstin Hanff, Arndt Quer, Ryuki Matsushita, Ryutaro Shiraishi, Maki Nagashima, Ayato Kimura, Kotaro Matsuishi, Makina Yabashi, Yoshihito Tanaka, Giorgio Rossi, Tetsuya Ishikawa, Kai Rossnagel, Masaki Oura

**Affiliations:** 1 Institut für Experimentelle und Angewandte Physik, Christian-Albrechts-Universität zu Kiel, 24098 Kiel, Germany; 2 RIKEN SPring-8 Center, 1-1-1 Kouto, Sayo-cho, Sayo-gun, Hyogo 679-5148, Japan; 3 Toyota Technological Institute, Nagoya 468-8511, Japan; 4 Synchrotron Light Application Center, Saga University, 1 Honjo, Saga 840-8502, Japan; 5 JASRI, 1-1-1 Kouto, Sayo-cho, Sayo-gun, Hyogo 679-5198, Japan; 6 Graduate School of Materials Science, University of Hyogo, Kamigori-cho, Ako-gun, Hyogo 678-1297, Japan; 7 Dipartimento di Fisica, Università degli Studi di Milano, Via Celoria 16, 20133 Milano, Italy

## Abstract

Time-resolved hard X-ray photoelectron spectroscopy (trHAXPES) using microfocused X-ray free-electron laser (XFEL, hν = 8 keV) pulses as a probe and infrared laser pulses (hν = 1.55 eV) as a pump is employed to determine intrinsic charge-carrier recombination dynamics in La:SrTiO_3_. By means of a combination of experiments and numerical *N*-body simulations, we first develop a simple approach to characterize and decrease XFEL-induced vacuum space-charge effects, which otherwise pose a serious limitation to spectroscopy experiments. We then show that, using an analytical mean-field model, vacuum space-charge effects can be counteracted by pump laser-induced photoholes at high excitation densities. This provides us a method to separate vacuum space-charge effects from the intrinsic charge-carrier recombination dynamics in the time domain. Our trHAXPES results thus open a route to studies of intrinsic charge-carrier dynamics on picosecond time scales with lateral spatial resolution on the micrometer scale.

X-ray-based solid-state photoelectron spectroscopy is a powerful tool for the investigation of electronic properties in condensed matter systems, combining element and atomic-site specificity with sensitivity to the chemical environment^1^,^2^. When photon energies in the hard X-ray regime are used, the technique becomes more bulk-sensitive and is referred to as hard X-ray photoelectron spectroscopy (HAXPES)^3^.

The recent development of new X-ray facilities such as X-ray free electrons lasers (XFELs) provides unprecedented capabilities in terms of the X-ray pulse duration available at high photon energies (down to a few tens of femtoseconds). Ultrashort hard X-ray pulses, in particular, make it possible to determine transient bulk electronic structure dynamics by means of time-resolved HAXPES (trHAXPES) experiments through a pump-probe scheme^4^,^5^,^6^. By simultaneously exploiting the possibility of focusing the photon beam down to a spot size of a few tens of micrometers or even less, spatial resolution can be added to the time-domain information, thereby opening a novel path to time-resolved studies of, e.g., vertical heterostructures, inhomogeneously doped materials, or micrometer-sized samples.

A fundamental limitation to all variants of time-resolved photoelectron spectroscopy are vacuum space-charge effects arising from the use of both ultrashort pump and probe pulses with high peak intensities^5^,^6^,^7^,^8^,^9^,^10^,^11^,^12^,^13^,^14^,^15^,^16^,^17^,^18^,^19^,^20^. Whenever more than one photoelectron is ejected into the vacuum by the absorption of a photon pulse, the Coulomb interaction among the electrons on their way to the detector becomes relevant, which can result in severe energy and momentum distortions of the detected photoelectron spectra. Although both pump and probe pulse-induced space-charge effects can be controlled experimentally and theoretically^6^,^13^,^16^,^18^,^19^, they are at present inevitable in trHAXPES experiments since the pulse intensities have to be sufficiently strong to overcome the low photoionization cross sections at the high photon energies used in combination with the low repetition rates of the photon sources currently available^4^,^5^,^6^. The use of a microfocused photon beam, resulting in a smaller focal spot size on the sample surface, can be expected to aggravate the space-charge problem further due to the higher density of emitted electrons per area and pulse^13^.

In this work, we show that, contrary to expectations, under suitable experimental geometry, trHAXPES using a microfocused XFEL beam can be used to probe intrinsic charge-carrier recombination dynamics in the electron-doped perovskite oxide La:SrTiO_3_ in the high pump excitation density regime. After a discussion of the general limitations arising from the use of a microfocused XFEL beam, namely sample ablation and XFEL-induced vacuum space-charge effects, we present (tr)HAXPES data of the Ti 1 *s* emission of La:SrTiO_3_ recorded as function of pulse energy, incidence angle, and time delay between a low-photon energy (1.55 eV) pump pulse and the XFEL pulse as a probe. We then perform two different types of calculations to analyze the data. First, by means of numerical *N*-body simulations, we quantify the impact of microfocusing on the space charge-induced spectral shift and broadening of the photoelectron kinetic energy distribution. The results show that for extremely low photon incidence angles (relative to the sample surface) space-charge distortions can be effectively reduced at an increasing detection count rate for a given photon flux, albeit at the expense of a reduction in spatial resolution in one dimension. Second, using an analytical mean-field model, we address the role of vacuum space-charge effects in the high pump excitation density regime. By comparison with the experimental results, we can deconvolve vacuum space-charge effects from intrinsic charge-carrier recombination dynamics on the picosecond to nanosecond time scale. The results reveal a complex three-staged dynamical behavior for positive time delays, i.e., when the probe pulse follows the pump pulse. The successful application of micro-trHAXPES beyond space-charge effects establishes a novel method to gain insight into spatially resolved ultrafast bulk electron dynamics, e.g., in complex materials, at buried interfaces, or in electronic devices under *in operando* conditions.

## Results and Discussion

### Fundamental limitations to XFEL-based micro-HAXPES

We start by discussing two general limitations of solid-state photoelectron spectroscopy arising from the use of a microfocused, ultrashort-pulsed photon source with high peak intensities: sample ablation^21^ and (probe-induced) vacuum space-charge effects^4^,^6^,^16^,^17^.

**Figure 1:**
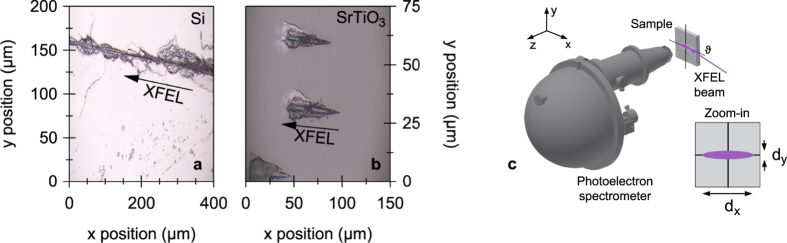
XFEL-induced sample ablation and experimental geometry. Radiation damages on the surfaces of Si (**a**) and La-doped SrTiO_3_ (**b**) when operating with a microfocused XFEL beam at a fluence, i.e., pulse energy per area, of 175 Jcm^−2^. The direction of photon incidence is indicated by arrows. (**c**) The angle of photon incidence *ϑ* (relative to the sample surface) can be varied by sample rotation, resulting in a changed spot diameter *d*_*x*_ on the sample surface at a constant spot diameter *d*_*y*_. The axis of the entrance lens of the photoelectron spectrometer (*z* direction) was perpendicular to the direction of the photon beam (*x* direction) in all cases.

When using the unattenuated microfocused SACLA XFEL photon beam, fluences, i.e., pulse energies per area, of approximately 175 Jcm^−2^ are reached, which are well above the ablation threshold of the samples used, e.g., 80 Jcm^−2^ in the case of silicon^21^, resulting in severe sample damage [Fig. 1(a,b)]. The magnitude of the ablation threshold fluence, *F*_abl_, for the single crystal La:SrTiO_3_ sample used in the present study can be estimated as 90 Jcm^−2^ < *F*_abl_ < 175 Jcm^−2^. This type of radiation damage typically does not arise in experiments with an unfocussed XFEL beam (spot diameter ~700 *μ*m)^4^,^5^,^6^.

**Figure 2:**
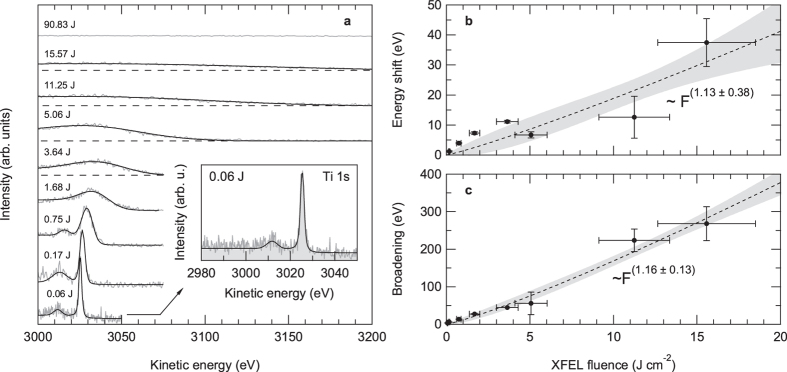
Space-charge effects in XFEL-based micro-HAXPES. (**a**) Evolution of the Ti 1 *s* core-level photoemission spectrum of La-doped SrTiO_3_ as a function of XFEL fluence (*hν* ≈ 8 keV). All fluences given are pulse energies per cm^2^. (**b**) Spectral shift and (**c**) spectral broadening of the Ti 1 *s* core-level emission of SrTiO_3_ as a function of XFEL fluence. The filled symbols are experimental data. The dashed line is a power-law fit to the data, 68.3% confidence bands are indicated by gray filling.

However, in order to perform photoelectron spectroscopy experiments, the average pulse energy has to be reduced further than just below the sample ablation threshold. This is because of vacuum space-charge effects, which can result in severe distortions of the recorded energy distribution curves. Figure 2(a) shows the evolution of the measured Ti 1 *s* HAXPES spectra of La:SrTiO_3_ as a function of the average XFEL pulse energy. These data were recorded at a photon incidence angle of about 1° relative to the sample surface. The applied mean fluences ranged from 0.06 Jcm^−2^ up to 90.83 Jcm^−2^. The latter value corresponds to a beam attenuation of 52%. With increasing XFEL pulse energies, the spectral distributions become broadened and shifted towards higher kinetic energies, until at the highest applied pulse energies no spectral features can be recognized anymore.

For a quantification of the observed space-charge effects, we have fitted the experimental data using Voigt profiles after subtraction of a Shirley-type background [Fig. 2(a)]. The extracted spectral shift and broadening are shown in Fig. 2(b,c). The space-charge broadenings were calculated as 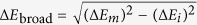, where Δ*E*_*m*_ is the measured, broadened (Gaussian) FWHM of the respective energy distribution curve and Δ*E*_*i*_ = 1.43 eV is the ‘intrinsic’ linewidth as determined by high-resolution HAXPES experiments at the same photon energy^4^ convoluted with the instrumental energy resolution (~1.25 eV FWHM). The data reveal the behavior known from previous XFEL photoemission experiments^6^ and predicted by numerical *N*-body simulations^6^,^15^: near-to-linear dependencies of the spectral shift and broadening as a function of the average fluence or, equivalently, the number of excited photoelectrons *N*. The fitted linear slopes are (1.98 ± 0.26) eV/(Jcm^−2^) and (17.48 ± 0.96) eV/(Jcm^−2^) for the spectral shift and broadening, respectively. Thus, to perform photoelectron spectroscopy experiments with a microfocused XFEL beam, or more precisely, to obtain spectral widths where the spectral broadening is smaller than the intrinsic linewidth, the available photon intensity has to be reduced by at least 3 orders of magnitude resulting in prolonged acquisition times of 40 to 60 minutes in comparison to measurements with a less attenuated beam (however, at a repetition rate of only 30 Hz).

### Incidence angle-dependence of probe pulse-induced space-charge effects

HAXPES experiments generally suffer from notoriously low photoionization cross sections at the typically used photon energies (6–8 keV) and thus comparably low detection count rates even at high-repetition-rate synchrotron radiation sources. A common approach to drastically increase the photoemission signal for a given photon flux is to measure in a grazing incidence geometry, i.e., to decrease the photon incidence angle relative to the surface while collecting photoelectrons in normal emission^22^.

**Figure 3:**
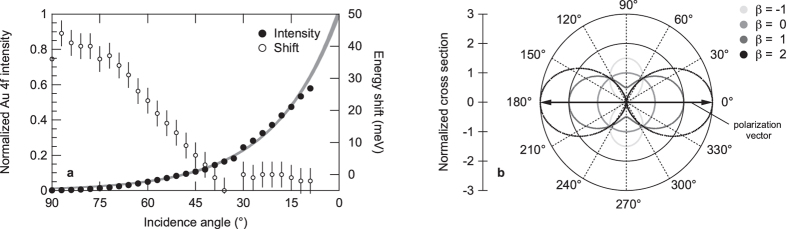
Photoelectron spectroscopy at grazing photon incidence. (**a**) Normalized photoemission signal of the Au 4*f* core-level spectra measured at BL17SU of SPring-8 (*hν* = 600 eV; *β* = 0.7075^23^) and corresponding space charge-induced spectral shift as a function of incidence angle. The solid gray line is a phenomenological fit to the experimental data. (**b**) Angular distribution of photoelectrons from free atoms. For a positive asymmetry parameter *β*, the photoemission intensities have a maximum at the direction of the polarization vector, i.e., when measuring in a grazing incidence and normal emission geometry^22^,^23^.

One underlying factor is the angle dependence of the photoionization cross section as sketched in Fig. 3(b). When linearly polarized light is used as an excitation source, the photoionization cross section of the examined material shows an angular distribution depending on the so-called asymmetry parameter *β*^22^,^23^. For HAXPES, almost all subshells have positive *β* values^23^. Thus, the number of detected photoelectrons reaches a maximum in the direction parallel to the polarization vector, i.e., when measuring in a grazing incidence and normal emission geometry. Another factor is the reduced light penetration depth at grazing incidence, which results in an increased number of excited electrons with an inelastic mean free path longer than their escape depth. By further reducing the photon incidence angle to a fraction of a degree, one could additionally reach surface sensitivity by exploiting the critical angle for total external reflection.

However, at first glance somewhat counterintuitively, the photoemission data, shown here for the example of the Au 4*f* core-level emission of gold (*β* = 0.7075^23^) measured with soft X-ray photoelectron spectroscopy, not only exhibit a drastic increase in photoemission intensity, i.e., number of ejected photoelectrons, with decreasing incidence angle, but also a decrease in the measured space charge-induced spectral shift [Fig. 3(a)].

In the following, we examine the impact of the relevant incidence angle-dependent experimental parameters on the space charge-induced spectral shift by means of numerical *N*-body simulations. The three most important parameters are: (i) the shape, i.e., eccentricity *ε* of the elliptical spot profile, (ii) the horizontal spot diameter *d*_*x*_ and the related change in spot area, as well as (iii) the number of emitted photoelectrons *N*.

**Figure 4:**
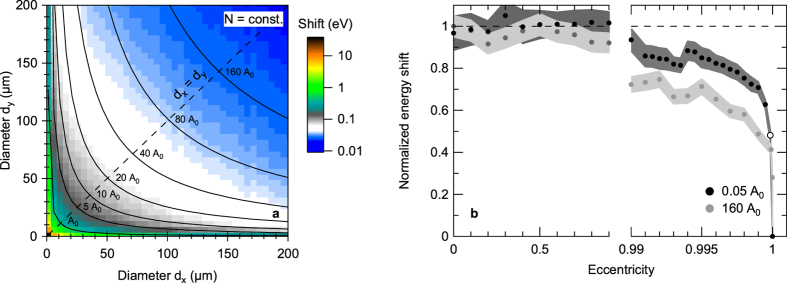
Influence of the beam footprint on space-charge effects in XFEL-based micro-HAXPES. (**a**) Simulated spectral shift for a constant number of photoelectrons as a function of horizontal and vertical spot diameter (*E*_kin_ = 3025 eV; Δ*t* = 10 fs; *N* = 1000). Black lines indicate beam footprints with the same size (*A*_0_ = 100 *μ*m^2^). (**b**) Spectral shift along lines of the same spot size. With increasing eccentricity of the beam spot the spectral shift decreases drastically when reaching an extremely elliptical regime although the number of electrons per area remains constant. The open circle marks the experimental geometry used in this study, i.e., a photon incidence angle of 1° relative to the surface plane (*ε* = 0.99851 at *d*_*y*_ = 2.5 *μ*m).

Figure 4(a) shows the simulated space-charge shift for a constant number of photoelectrons as a function of the horizontal and vertical spot diameter (for *E*_kin_ = 3025 eV and Δ*t* = 10 fs). Unsurprisingly, the spectral shift decreases when increasing the spot area by elongating one or both of the spot diameters *d*_*x*,*y*_, i.e., when decreasing the number of photoelectrons per area. More crucially, however, the spectral shift also decreases at a constant number of photoelectrons per area when the spot eccentricity 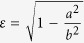, where *a* is the minor and *b* the major axis of the ellipse, is increased beyond a value of *ε* > 0.99 [Fig. 4(b)]. For a microfocused elliptical beam of 100 *μ*m^2^ spot size, for example, this eccentricity corresponds to spot dimensions of (4.25 × 30) *μ*m^2^, i.e., 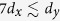. Thus, space-charge effects can indeed be reduced at a given photon flux and spot size by choosing an elliptical instead of a circular spot profile. Intuitively, the effect can be understood as a transition of the photoelectron cloud from a compact two-dimensional disk of charge to an elongated quasi-one-dimensional chain of charge, which comes along with a decreased Coulomb potential.

**Figure 5:**
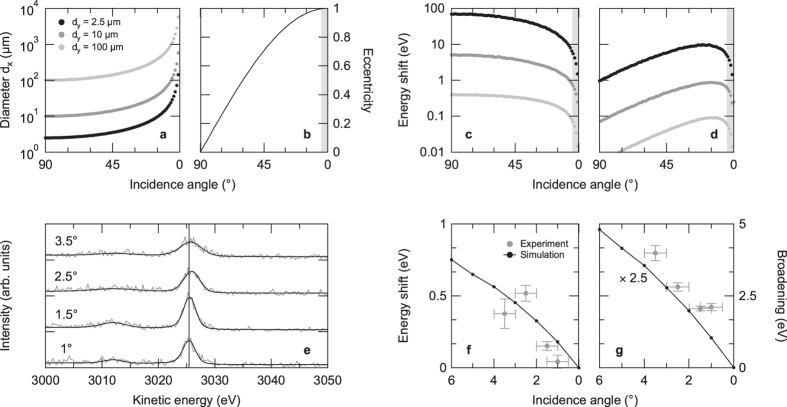
Comparison of simulated and measured space-charge shift as a function of photon incidence angle. (**a**) Horizontal spot diameter *d*_*x*_ and (**b**) corresponding eccentricity as a function of incidence angle (relative to the surface) starting from a circular spatial distribution. The shaded part in (**b**) [and also in (**c**) and (**d**)] corresponds to an eccentricity *ε* > 0.997. (**c**) Simulated space-charge shift, including the change in horizontal spot diameter and eccentricity, as a function of incidence angle. (**d**) Simulated space-charge shift as a function of incidence angle multiplied by the related non-linear change in the number of ejected photoelectrons [Fig. 3(a)]. (**e**) Evolution of the Ti 1 *s* emission of La:SrTiO_3_ as a function of photon incidence angle relative to the surface (*hν* ≈ 8 keV). Upon more grazing incidence, the measured space charge-induced spectral shift and broadening decrease. The vertical line marks the peak position at an incidence angle of 1°. (**f**,**g**) Simulated versus measured spectral shift and broadening of the Ti 1 *s* emission as a function of incidence angle. Note that the simulations include the angle-dependent change in the number of excited photoelectrons and that the simulated broadening is multiplied by a factor of 2.5 to match the experimental results.

Given the above approach, we can now use it to optimize the conditions in our micro-HAXPES experiments. Figure 5(a,b) shows the calculated elongation of the horizontal spot diameter as a function of incidence angle, starting from a circular Gaussian-shaped spot profile, as well as the corresponding change in eccentricity for three different initial spot diameters. The space charge-induced spectral shift at a spot diameter of *d*_*y*_ = 2.5 *μ*m is reduced by >20% (with respect to normal photon incidence) when an eccentricity of *ε* > 0.997 is reached, corresponding to a photon incidence angle of 4.5° relative to the surface plane. This is directly reflected in a drastic decrease of the simulated space-charge shift as a function of incidence angle by one order of magnitude (when keeping the number of photoelectrons constant) [Fig. 5(c)]. Importantly, even when the non-linear increase in the number of ejected photoelectrons at low photon incidence angles [Fig. 3(a)] is included, the expected increase in space charge-induced shift due to its near-to-linear dependence on the number of ejected photoelectrons is compensated by the increase in spot area and eccentricity. Thus, overall, using a grazing incidence geometry should result in a decrease of space-charge effects at an increasing number of emitted photoelectrons [Fig. 5(d)].

To check this prediction, we have experimentally determined the angular dependence of the space-charge shift and broadening by analyzing the Ti 1 *s* emission of La:SrTiO_3_ as a function of photon incidence angle in a range of 3.5° to 1° [Fig. 5(e)]. For a quantification of the observed spectral distortions, we have fitted the experimental data with Voigt profiles after subtraction of a Shirley-type background. Best fits are included in Fig. 5(e). The (Gaussian) broadening was calculated as described above. The extracted spectral shifts and broadenings are presented in Fig. 5(f,g): With decreasing photon incidence angle the measured spectral broadening as well as the space charge-induced shift in kinetic energy decrease, in good qualitative agreement with the results of our numerical *N*-body simulations. Note that the numerical simulations underestimate the measured spectral broadening by a factor of about 2.5. We tentatively attribute this discrepancy to the deviations of the experimental angle and energy distributions from being isotropic and monoenergetic^6^, respectively. Any anisotropy in the photoelectron emission^9^,^10^ as well as the inclusion of a secondary photoelectron background^15^ or other photoemission lines^17^ in the initial spectral distribution may give rise to enhanced spectral broadenings at a given number of excited photoelectrons.

Summing up, the above results establish a simple experimental approach to reduce both data acquisition time as well as space-charge effects in (micro-)trHAXPES experiments at ultrashort-pulsed XFEL facilities with respect to experiments conducted in a non-grazing photon incidence geometry.

### Space-charge and charge-carrier recombination dynamics in trHAXPES

Finally, we exploit the novel approach and investigate the 1.55-eV-pump laser-induced dynamics of the Ti 1 *s* emission of La-doped SrTiO_3_ as probed by the 8 keV XFEL radiation. The pump laser was set to a fluence of 30 mJcm^−2^ and the XFEL probe fluence on the sample was 0.17 Jcm^−2^.

Importantly, we note that in comparison to our previous trHAXPES experiments on La:SrTiO_3_, in which an unfocused XFEL photon beam was used at a pump and probe photon incidence angle of *ϑ* ≈ 15° (relative to the sample surface)^6^, the reduced photon incidence angle of *ϑ* ≈ 1°–1.5° leads to an up to 15-fold reduction of the effective XFEL penetration depth, 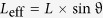, where *L*(8 keV, *ϑ* = 90°) ≈ 5.5 nm^24^. By contrast, the pump laser penetration depth, which is assumed to be in the order of 52 nm as reported for the related material SrRuO_3_^25^, does not change significantly upon reduction of the incidence angle^26^. Hence, whereas similar incident pump laser fluences and thus similar excitation densities were used in the two experiments, the present grazing-incidence measurements enable us to be more sensitive to surface effects due to the drastically decreased effective XFEL probing depth.

**Figure 6:**
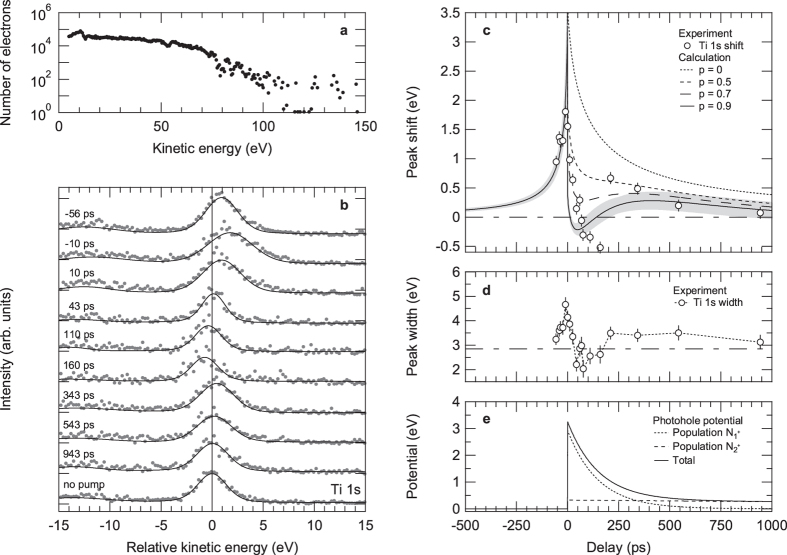
Tracking pump laser-induced dynamics in La:SrTiO_3_. (**a**) Low-energy photoemission spectrum of La:SrTiO_3_ for a pump fluence of 30 mJcm^−2^. The photoemission intensity is normalized to the total number of photoelectrons. (**b**) Evolution of selected Ti 1 *s* core-level photoemission spectra of La:SrTiO_3_ as a function of pump-probe delay at a pump fluence of 30 mJcm^−2^ (*hν* ≈ 8 keV). The vertical line marks the peak position for a blocked pump beam (*E*_0_ = 3026.31 eV). The maximum pump laser-induced shift towards higher kinetic energies is observed at time zero when pump and probe pulses overlap in time. (**c**) Spectral shift of the Ti 1 *s* emission as a function of pump-probe delay. The open circles are experimental data; the lines are from an analytical model considering different fractions of stationary photoholes *p* inside the sample. Gray shadings represent the error bars of the calculation assuming a charge fraction of *p* = 0.9. (**d**) Measured Gaussian width of the Ti 1 *s* emission as a function of pump-probe delay. The dashed horizontal line marks the peak width for a blocked pump beam. (**e**) Photohole recombination dynamics inside the sample extracted from the mean-field calculations (*p* = 0.9 photoholes per pump electron included). The photohole population (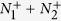) as well as the related electrostatic potential are assumed to decay bi-exponentially as a function of time with time constants *τ*_1_ = 150 ps and *τ*_2_ = 5 ns.

Figure 6(b) shows the experimental spectra for various pump-probe delays together with the best fits using Voigt profiles on a Shirley-type background. The zero of the energy axis is defined by the position of the Ti 1 *s* emission at a blocked pump beam. The maximum positive kinetic energy shift and broadening are observed at time zero, when pump and probe pulses overlap in time^5^,^6^,^16^,^17^,^20^.

The relaxation dynamics of the extracted shift in kinetic energy as well as the spectral width show a distinctly different character for positive and negative delays [Fig. 6(c,d)]. For negative delays both decay on a 100 picosecond time scale, whereas for positive delays three-staged dynamics can be observed. The measured spectral shift and broadening, first, decrease within a few tens of picoseconds toward a minimum at kinetic energies and spectral widths, respectively, lower than the mean values of the unpumped spectrum, before, second, recovering toward positive shift and broadening values and, finally, relaxing back into equilibrium on a nanosecond time scale.

To understand the origin of this dynamics, we first present the results of mean-field model calculations neglecting the possible presence of (quasi-)stationary photoholes at the surface (*p* = 0 photoholes per pump electron). Long-living photoholes near the surface may in principle arise from pump laser-induced multiphoton electron emission into vacuum, electron-hole separation following internal photoexcitation in a space-charge layer beneath the surface, or a combination of both. We note that distinguishing between these processes is principally difficult because they manifest similarly in the measured spectral photoelectron distributions^20^,^27^,^28^,^29^. To identify the dominant mechanism, one could for example exploit the expected differences in the pump fluence dependence of the effects: While surface photovoltage effects typically saturate at high pump fluences^30^, the number of photoholes left behind from multiphoton photoemission and the corresponding attractive Coulomb potential should increase nonlinearly as a function of pump fluence^6^,^31^. Moreover, by choosing a hole-doped sample, possible transient photovoltages should change sign under the same measurement conditions^32^. Such investigations are, however, beyond the scope of the present work.

Figure 6(c) shows the time dependence of the Ti 1 *s* shift extracted from the experimental data in comparison to the calculated results. To reproduce the maximum positive kinetic energy shift as well as the negative delay dynamics, the number of pump electrons had to be set to 3.5 ⋅ 10^6^. The beam spot diameters as well as the mean pump- and probe-electron velocities, on the other hand, were taken from the experiment. The mean kinetic energy of the emitted pump electrons was determined from the measured energy distribution curve displayed in Fig. 6(a).

When neglecting any possible influence of pump laser-induced photohole effects at the surface (*p* = 0) and only accounting for pump laser-induced space-charge effects in vacuum, the model can *not* reproduce the observed three-staged dynamics for positive time delays [Fig. 6(c)]. However, when we take into account stationary photohole states (*p *> 0) as well as possible bi-exponential electron-hole recombination inside the strongly electron-doped SrTiO_3_ sample (with time constants *τ*_1_ and *τ*_2_), the model can qualitatively describe the observed delay dependence of the spectral shift (see Sec. Methods). In fact, the calculations give a successively better agreement with the experimental data for an increasing number of photoholes at the surface [Fig. 6(c)]. The best agreement can be found if *p* = 0.9 stationary photoholes per excited pump electron are assumed.

We note that in our simple 1D model the position of the minimum at kinetic energies lower than the mean value of the unpumped spectrum is mainly determined by the time constant *τ*_1_, which, however, at the same time defines the recovery rate toward positive kinetic energy shift values. The chosen value of the time constant *τ*_1_ appears to be the best fit between both stages. The subsequent relaxation into equilibrium is mainly governed by the value of the time constant *τ*_2_. The charge-carrier recombination time constants of *τ*_1_ = (150 ± 20) ps and *τ*_2_ = (5 ± 0.5) ns chosen to reproduce the observed dynamics [Fig. 6(e)] as well as the assumed charge population ratio 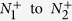 of (9 ± 0.5) to 1 are in good agreement with the findings of time-resolved photoluminescence experiments in the high excitation density regime on undoped SrTiO_3_ samples^33^,^34^. The microscopic origin of these recombination processes, however, remains an interesting subject for further investigations. We note that similar effects, i.e., negative shifts in kinetic energy due to the influence of photoholes in the high excitation density regime, have recently also been observed in time-resolved extreme ultraviolet photoelectron spectroscopy of solutions^35^.

In view of the simplicity of the model, the agreement between calculated and measured results is remarkable. Our simple mean-field model can be used to deconvolve pump laser-induced extrinsic space-charge dynamics and intrinsic charge-carrier recombination dynamics in high kinetic energy photoelectron spectroscopy. The combined experimental and theoretical approach thus establishes trHAXPES as a novel spectroscopic tool for determining electron recombination dynamics with bulk sensitivity.

In conclusion, we have realized a successful application of trHAXPES using a microfocused XFEL photon beam. To this end, we first determined the impact that focal spot sizes of a few micrometers have on the inevitable space charge-induced spectral distortions of the recorded photoemission spectra. By means of numerical simulations and experiments, we found lowering the photon incidence angle to be a viable approach to reduce XFEL-induced space-charge effects at an increased detection count rate, albeit at the expense of a loss of spatial resolution in one dimension. Importantly, by an application of micro-trHAXPES to electron-doped SrTiO_3_ and the use of a simple analytical mean-field model, we could then deconvolve extrinsic vacuum space-charge effects from intrinsic charge-carrier recombination dynamics in the time domain. Our results reveal a bi-exponential decay of the pump excitation-induced photoholes on a picosecond to nanosecond time scale, in good agreement with the findings of time-resolved photoluminescence studies. Hence, these results establish trHAXPES with lateral spatial resolution on the micrometer scale as a novel technique to determine intrinsic spatiotemporal charge-carrier dynamics on ultrafast time scales.

## Methods

### Experimental techniques

(Tr)HAXPES experiments were performed at beamline 2 (experimental hutch 3 providing a microfocused beam) of the SACLA XFEL facility at SPring-8^36^,^37^ using ultrashort (Δ*t* ≈ 10 fs), quasi-monochromatic (Δ*E* ≈ 1 eV) XFEL pulses with a photon energy of ~8 keV at a repetition rate of 30 Hz. The XFEL pulse timing jitter was at maximum ~250 fs. The average XFEL fluence was about 175 Jcm^−2^, corresponding to ~4.1 × 10^9^ photons per pulse, with 10% fluctuation over 30 shots. The pulse energy at the sample was adjusted by inserting Si and Al attenuators of varying thickness into the beam. Typical attenuation factors were in the range of up to 1000–2500. All photoemission spectra were recorded using a Scienta R4000-10 kV electron analyzer. For the (tr)HAXPES experiments the pass energy was set to 200 eV at an entrance slit width of 1.5 mm resulting in a nominal analyzer energy resolution of 0.75 eV and thus a total experimental energy resolution of about 1.25 eV. The typical data acquisition time for one spectrum was about 40–60 minutes.

For the time-resolved pump-probe photoemission studies, the XFEL probe pulses were complemented by synchronized optical pump pulses delivered by a Ti:Sapphire amplifier system with a photon energy of 1.55 eV, a pulse length of Δ*t* ≈ 40 fs, and incident fluences of up to 30 mJcm^−2^. The effective probe and pump beam spot sizes on the sample (full width at half maximum) were about 2.5 × (40–145) *μ*m^2^ and 190 × (2300–5000) *μ*m^2^, respectively, depending on the photon incidence angle, which was chosen in a range of 3.5° to 1° (relative to the sample surface). In this scheme, the horizontal pump spot diameter was limited by the sample size. Pump and probe beam hit the sample quasi-collinearly with an angle of 1° between the beams. In this experimental geometry, the relative delays between pump and probe pulses are maintained when measuring in a grazing photon incidence geometry. However, the optical path for the photon pulses increases along the major axis of the spot profiles and the photoelectron excitation process is thus spread in time (by <2 ps along the footprint of the XFEL beam). The temporal overlap of the pulses was determined using an ultrafast photodiode with a rise time of 30 ps and the pump-probe time delay was adjusted by using an optical delayline. As single crystal samples, 5% La-doped SrTiO_3_ as well as undoped Si were chosen. The equilibrium sample temperature during all experiments was set to 300 K.

Complementary soft X-ray PES experiments were conducted on a polycrystalline gold sample at the undulator beamline BL17SU of SPring-8 using a photon energy of 600 eV at a total energy resolution of 200 meV. Soft X-ray PES and (tr)HAXPES experiments were carried out using the same experimental setup with the photoelectron emission direction being perpendicular to the direction of photon incidence.

### Numerical *N*-body simulations

For the numerical simulation of the XFEL pulse-induced space-charge effects, we used a modified Barnes-Hut treecode algorithm for Coulomb force calculation and a leap-frog integration scheme to solve the equations of motion. The details of these numerical *N*-body simulations are described elsewhere^10^,^13^. The approach is to gradually evolve an *N*-electron distribution in front of the sample surface, assuming initial Gaussian spectral, temporal, and spatial profiles. The isotropically emitted photoelectrons interact on their way to the detector. The evolution of the photoelectron distribution is calculated until Coulomb forces become negligible, which typically occurs after a few nanoseconds. The space charge-induced distortions of the kinetic energy distribution, i.e., spectral shifts and broadenings, are quantified by fitting a Gaussian profile to the final spectral distribution of the photoelectrons reaching the detector. For these calculations, we neglected a possible presence of mirror charges below the sample surface. For all simulations, the initial kinetic energy of the initially isotropic photoelectron distribution was set to *E*_*i*_ = 3025 eV at a Gaussian FWHM of 1.45 eV, correponding to the “intrinsic” linewidth of the Ti 1 *s* emission of La:SrTiO_3_ at the used photon energy^4^. The Gaussian temporal profile was assumed to have a FWHM of 10 fs in all cases.

### Mean-field model

**Figure 7:**
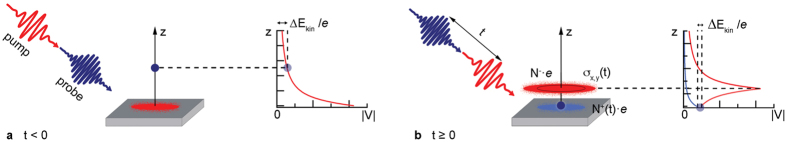
Mean-field model used to reproduce the dynamics of the pump pulse-induced spectral shift. For both (**a**) negative and (**b**) positive delays *t* the *N*^−^-electron cloud emitted by the pump pulse is modeled by a Gaussian charge distribution with standard deviations *σ*_*x*,*y*,*z*_ and the probe electron is allowed to move only in the direction of normal emission (*z* direction). Long-living photohole states at the surface (*z* = 0), *N*^+^(*t*), are modeled as the positive mirror image of the pump electron charge distribution directly after its birth. For negative delays *t*, these positive charges are considered to be completely screened by the pump electron disk, while for positive delays a bi-exponential decay of the number of photoholes is assumed. The final pump laser-induced shift in kinetic energy, Δ*E*_kin_, is calculated as the probe-electron potential energy *eV* directly after the birth of the pump electrons (**a**) or the probe electron (**b**).

To reproduce the measured pump-probe delay dependence of the pump laser-induced spectral shift, we extended a simple mean-field model, which was successfully used to describe pump laser-induced space-charge effects in trHAXPES experiments^5^,^6^. The basic assumption of this model, sketched in Fig. 7, is to approximate the electron cloud excited by the pump pulse as a Gaussian charge distribution moving at the average pump-electron velocity *v*_pump_ in the direction normal to the surface (*z* direction). The on-axis potential of such a charge distribution can be calculated numerically as^38^,^39^


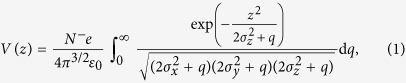


where *N*^−^ is the number of electrons, *e* the elementary charge, *ε*_0_ the electric constant, *σ*_*x*,*y*,*z*_ are the respective standard deviations of the Gaussian charge distribution, and *z* is the axial distance from its center. The radial expansion of the electron cloud is incorporated by assuming a two-dimensional expansion with the mean pump-electron velocity.

To account for long-living photoholes at the surface (*z* = 0) arising from multiphoton electron emission or separation of photoexcited electron-hole pairs in a surface space-charge layer, an additional positive charge distribution of *N*^+^ = *p* ⋅ *N*^−^ charge carriers, *p* ∈ [0, 1], is introduced, equaling the shape of the pump electron charge distribution directly after its birth. Electron-hole recombination mechanisms inside the sample, such as nonlinear Auger recombination, single-carrier trapping, or other microscopic carrier recombination processes, are included by phenomenologically assuming a bi-exponential decay of the number of positive charge carriers, 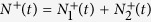, based on the results of time-resolved photoluminescence studies of SrTiO_3_ in the high excitation density regime^33^,^34^,^40^,^41^.

The inital *z* seperation between the centers of gravity of the probe electron and the pump electron spatial distribution depends on the pump-probe delay *t* and is given by


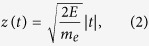


where *m*_*e*_ is the electron mass and *E* is the probe-electron kinetic energy for *t* < 0 (probe pulse before pump pulse) or the average pump-electron kinetic energy for *t* ≥ 0, respectively. The final change in kinetic energy of the probe electron, Δ*E*_kin_(*t*), equals the inital total potential energy of the probe electron, *e*(|*V*^−^[*z*(*t*)]| − |*V*^+^[*z*(*t*)]|), where *V*^−^[*z*(*t*)] is the potential of the pump electron distribution and *V*^+^[*z*(*t*)] the potential of the photohole distribution. For simplicity, the photohole distribution is assumed to be completely screened by the pump-electron disk for negative delays, i.e., *V*^+^(*t* ≤ 0) = 0.

## Additional Information

**How to cite this article**: Oloff, L.-P. *et al*. Time-resolved HAXPES using a microfocused XFEL beam: From vacuum space-charge effects to intrinsic charge-carrier recombination dynamics. *Sci. Rep.* **6**, 35087; doi: 10.1038/srep35087 (2016).

## References

[b1] Fahlmann A., Hamrin K., Hedman J., Nordberg R., Nordling C. & Siegbahn K. Electron spectroscopy and chemical binding. Nature 210, 4 (1966).

[b2] Lindau I., Pianetta P., Doniach S. & Spicer W. X-ray photoemission spectroscopy. Nature 250, 214 (1974).

[b3] Woicik J. C. In Hard x-ray photoelectron spectroscopy (HAXPES) (ed. Woicik J. C.) (Springer International Publishing, 2015).

[b4] Oura M. . Development of a single-shot CCD-based data acquisition system for time-resolved x-ray photoelectron spectroscopy at an x-ray free-electron laser facility. J. Synchrotron Rad. 21, 183 (2014). 10.1107/S1600577513028233 PMC4421850 24365935

[b5] Oura M. . Electron dynamics probed by time-resolved hard x-ray photoelectron spectroscopy. Trans. Mat. Res. Soc. Jpn. 39, 469 (2014).

[b6] Oloff L.-P. . Time-resolved HAXPES at SACLA: probe and pump pulse-induced space-charge effects. New J. Phys. 16, 123045 (2014).

[b7] Zhou X. J. . Space charge effect and mirror charge effect in photoemission spectroscopy. J. Electron Spectrosc. Relat. Phenom. 142, 27 (2005).

[b8] Passlack S., Mathias S., Andreyev O., Mittnacht D., Aeschlimann M. & Bauer M. Space charge effects in photoemission with a low repetition, high intensity femtosecond laser source. J. Appl. Phys. 100, 024912 (2006).

[b9] Pietzsch A. . Towards time resolved core level photoelectron spectroscopy with femtosecond x-ray free-electron lasers. New J. Phys. 10, 033004 (2008).

[b10] Hellmann S., Rossnagel K., Marczynski-Bühlow M. & Kipp L. Vacuum space-charge effects in solid-state photoemission. Phys. Rev. B 79, 035402 (2009).

[b11] Graf J. . Vacuum space charge effect in laser-based solid-state photoemission spectroscopy. J. Appl. Phys. 107, 014912 (2010).

[b12] Faure J. . Full characterization and optimization of a femtosecond ultraviolet laser source for time and angle-resolved photoemission on solid surfaces. Rev. Sci. Instrum. 83, 043109 (2012). 22559517 10.1063/1.3700190

[b13] Hellmann S., Ott T., Kipp L. & Rossnagel K. Vacuum space-charge effects in nano-ARPES. Phys. Rev. B 85, 075109 (2012).

[b14] Schönhense G. . Correction of the deterministic part of space–charge interaction in momentum microscopy of charged particles. Ultramicroscopy 159, 488 (2015). 26051657 10.1016/j.ultramic.2015.05.015

[b15] Verna A., Greco G., Lollobrigida V., Offi F. & Stefani G. Space-charge effects in high-energy photoemission. J. Electron Spectrosc. Relat. Phenom. 209, 14 (2016).

[b16] Hellmann S. . Time-resolved x-ray photoelectron spectroscopy at FLASH. New J. Phys. 14, 013062 (2012).

[b17] Dell’Angela M. . Vacuum space charge effects in sub-picosecond soft X-ray photoemission on a molecular adsorbate layer. Struct. Dyn. 2, 025101 (2015). 26798795 10.1063/1.4914892 PMC4711610

[b18] Leuenberger D., Yanagisawa H., Roth S., Osterwalder J. & Hengsberger M. Disentanglement of electron dynamics and space-charge effects in time-resolved photoemission from *h*-BN/Ni(111). Phys. Rev. B 84, 125107 (2011).

[b19] Oloff L.-P. . Pump laser-induced space-charge effects in HHG-driven time- and angle-resolved photoelectron spectroscopy. J. Appl. Phys. 119, 225106 (2016).

[b20] Ulstrup S. . Ramifications of optical pumping on the interpretation of time-resolved photoemission experiments on graphene. J. Electron Spectrosc. Relat. Phenom. 200, 340 (2015).

[b21] Koyama T. . Damage study of optical substrates using 1-μm-focusing beam of hard x-ray free-electron laser. J. Phys. Conf. Ser. 463, 012043 (2013).

[b22] Takata Y. . Development of hard x-ray photoelectron spectroscopy at BL29XU in SPring-8. Nucl. Instr. Meth. Phys. Res. A 547, 50 (2005).

[b23] Yeh J. J. & Lindau I. Atomic subshell photoionization cross sections and asymmetry parameters: 1 ≤ Z ≤ 103. At. Data Nucl. Data Tables 32, 1 (1985).

[b24] Dallera C. . Looking 100 Å deep into spatially inhomogeneous dilute systems with hard X-ray photoemission. Appl. Phys. Lett. 85, 4532 (2004).

[b25] Kostic P. . Non-Fermi-Liquid Behavior of SrRuO_3_: Evidence from Infrared Conductivity. Phys. Rev. Lett. 81, 2498 (1998).

[b26] Born M. & Wolf E. In Principles of Optics (ed. Born M. & Wolf E.) (Pergamon, 1980).

[b27] Widdra W. . Time-resolved core level photoemission: Surface photovoltage dynamics of the SiO_2_/Si(100) interface. Surf. Sci. 543, 87 (2003).

[b28] Yang S.-L., Sobota J. A., Kirchmann P. S. & Shen Z.-X. Electron propagation from a photo-excited surface: implications for time-resolved photoemission. Appl. Phys. A 116, 85 (2014).

[b29] Tanaka S.-I. Utility and constraint on the use of pump-probe photoelectron spectroscopy for detecting time-resolved surface photovoltage. J. Electron Spectrosc. Relat. Phenom. 185, 152 (2012).

[b30] SpencerB. F. . Time-resolved surface photovoltage measurements at *n*-type photovoltaic surfaces: Si(111) and ZnO( 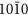 ). Phys. Rev. B 88, 195301 (2013).

[b31] Damascelli A., Gabetta G., Lumachi A., Fini L. & Parmigiani F., Multiphoton electron emission from Cu and W: An angle-resolved study. Phys. Rev. B 54, 6031 (1996). 10.1103/physrevb.54.6031 9986599

[b32] Siffalovic P., Drescher M. & Heinzman U. Femtosecond time-resolved core-level photoelectron spectroscopy tracking surface photovoltage transients on *p*–GaAs. Europhys. Lett. 60, 924 (2002).

[b33] Yasuda H. & Kanemitsu Y. Dynamics of nonlinear blue photoluminescence and Auger recombination in SrTiO_3_. Phys. Rev. B 77, 193202 (2008).

[b34] Rubano A., Paparo D., Miletto Granozio F., Scotti di Uccio U. & Marrucci L. Blue luminescence of SrTiO_3_ under intense optical excitation. J. Appl. Phys. 106, 103515 (2009).

[b35] Al-Obaidi R. . Ultrafast photoelectron spectroscopy of solutions: space-charge effect. New J. Phys. 17, 093016 (2015).

[b36] Ishikawa T. . A compact X-ray free-electron laser emitting in the sub-ångström region. Nat. Photon. 6, 540 (2012).

[b37] Tono K. . Beamline, experimental stations and photon beam diagnostics for the hard x-ray free electron laser of SACLA. New J. Phys. 15, 083035 (2013).

[b38] Kheifets S. Potential of a three-dimensional Gaussian bunch. PETRA Kurzmitteilung 119 (1976).

[b39] Takayama K. Potential of a 3-dimensional charge distribution. Fermilab Technical Memo 192 (1982).

[b40] Yamada Y., Yasuda H., Tayagaki T. & Kanemitsu Y. Photocarrier recombination dynamics in highly excited SrTiO_3_ studied by transient absorption and photoluminescence spectroscopy. Appl. Phys. Lett. 95, 121112 (2009).

[b41] Yamada Y. & Kanemitsu Y. Blue light emission from strongly photoexcited and electron-doped SrTiO_3_. J. Appl. Phys. 109, 102410 (2011).

